# A Retrospective Study of the Effectiveness of the AeroChamber Plus^®^ Flow-Vu^®^ Antistatic Valved Holding Chamber for Asthma Control

**DOI:** 10.1007/s41030-017-0047-1

**Published:** 2017-07-14

**Authors:** Chakkarin Burudpakdee, Vladimir Kushnarev, Dominic Coppolo, Jason A. Suggett

**Affiliations:** 1 QuintilesIMS, Fairfax, VA USA; 2 University of North Carolina at Charlotte, Charlotte, NC USA; 3 Trudell Medical International, London, ON Canada; 4 Monaghan Medical Corporation, Syracuse, NY USA

**Keywords:** Asthma, Exacerbations, Spacers, Valved holding chambers

## Abstract

**Introduction:**

Electrostatic charge in valved holding chambers (VHCs) may lead to inconsistent metered-dose inhaler (MDI) asthma drug delivery. We compared the AeroChamber Plus^®^ Flow Vu^®^ Antistatic Valved Holding Chamber (AC^+^FV AVHC) with non-antistatic control VHCs in terms of asthma exacerbations, resource use, and cost in an asthma population.

**Methods:**

Patients included in an adjudicated claims database with AC^+^FV AVHC or non-antistatic VHC (control VHC) use between 1/2010 and 8/2015 (index) who were treated with an inhaled corticosteroid (ICS) or a combination of an ICS and a long-acting β2 agonist MDI within 60 days before or after the index date, were diagnosed with asthma, and had ≥12 months of pre- and ≥30 days of post-index health plan enrollment were included. Cohorts were matched 1:1 using propensity scores. We compared incidence rates (IR) of exacerbation, time to first exacerbation using Kaplan–Meier survival analysis, occurrence of exacerbations, and healthcare resource use and costs using generalized linear models.

**Results:**

9325 patients in each cohort were identified. The IR of exacerbations per 100 person-days (95% CI) was significantly higher in the control VHC cohort than the AC^+^FV AVHC cohort [0.161 (0.150–0.172) vs. 0.137 (0.128–0.147)]. A higher proportion of exacerbation-free patients was observed in the AC^+^FV AVHC cohort. Among the 4293 patients in each cohort with ≥12 months of follow-up, AC^+^FV AVHC patients were found to be 10–12% less likely than control VHC patients to experience an exacerbation throughout the study period. A lower proportion of the AC^+^FV AVHC patients had an ED visit compared to the control VHC patients (10.8% vs. 12.4%). Exacerbation-related costs for the AC^+^FV AVHC cohort were 23%, 25%, 20%, and 12% lower than those for the control VHC cohort at 1, 6, 9, and 12 months, respectively.

**Conclusions:**

The AC^+^FV AVHC was associated with lower exacerbation rates, delayed time to first exacerbation, and lower exacerbation-related costs when compared to control non-antistatic VHCs.

## Introduction

Asthma is a common respiratory condition which affects approximately 7.4% of the US adult population and 8.6% of the US pediatric population [1]. The condition is characterized by airway inflammation which can lead to recurrent episodes of exacerbations and cause symptoms of wheezing, breathlessness, chest tightness, and coughing [2, 3]. These episodes of exacerbations often require treatment with a corticosteroid to reduce airway inflammation, and patients with worsening symptoms are commonly treated in the hospital or emergency department (ED) [4, 5]. In the US, asthma exacerbations result in 15 million outpatient visits, 2 million ED visits, and 500,000 inpatient admissions annually [5]. The annual direct and indirect cost of asthma care in the US is around $56 billion [6].

Medications administered by metered dose inhalers (MDIs), such as inhaled corticosteroids (ICS) and bronchodilators, are the mainstay of long-term control asthma treatment to prevent the occurrence of exacerbations [5, 7, 8]. However, patients with poor hand-breath actuation coordination may have difficulty in using MDIs properly and can remain vulnerable to exacerbations [5, 9]. Valved holding chambers (VHCs) are designed to reduce oropharyngeal deposition by substantially changing the aerodynamic particle size distribution of the inhaled aerosol, and can assist in improving drug delivery to the lungs by holding the aerosol plume of medication-loaded particles until the patient is ready to inhale, eliminating the need to carefully coordinate the timing of MDI actuation and inhalation [10, 11]. A recent study assessing the relationship between asthma control and patient ability to use their MDIs found that patients who used a spacer with their MDIs had significantly better asthma control compared to those who used MDIs alone [12]. However, the effectiveness of VHCs and other spacer devices can be adversely affected by electrostatic charge, a commonly reported cause of inconsistent medication delivery [13].

Valved holding chamber (VHC) devices made of antistatic materials offer a potential solution by reducing dose variability related to electrostatic charge, and may in theory help improve asthma control in patients who use VHCs to assist with aerosolized drug delivery [13, 14]. A laboratory investigation by Suggett et al. found that antistatic VHCs delivered more respirable medication from the MDI compared to non-antistatic VHCs, in part due to the antistatic nature of the materials [15]. However, no clinical or real-world studies have evaluated the effects of an antistatic VHC on asthma outcomes. The AeroChamber Plus^®^ Flow Vu^®^ Antistatic Valved Holding Chamber (AC^+^FV AVHC) is an antistatic VHC designed to be used with pressurized MDIs to administer aerosolized medication in patients who may have difficulty with the coordination and control involved in using MDIs correctly. An additional benefit of the AC^+^FV AVHC is the incorporation of an inspiratory flow indicator (IFI) for the patient/caregiver to observe effective inhalation. The IFI provides real-time feedback confirming an effective inhalation and ensures that there are no leakages of ambient air into the space between facemask and face that could prevent medication delivery altogether [16, 17]. The objective of this study was to compare the effects of the antistatic AC^+^FV AVHC and non-antistatic control VHCs on treatment outcomes, resource use, and healthcare costs in a real-world asthma population.

## Methods

This retrospective database study used data from the QuintilesIMS Real-World Data Adjudicated Claims Database (formerly known as PharMetrics Plus). The database contains adjudicated medical and pharmacy claims for more than 150 million US health plan members from 2006 onwards. The data are patient-level, longitudinal, and representative of the US commercially insured population. The database covers 90% of US hospitals, 80% of all US doctors’ offices, and relate to 85% of Fortune 100 employees. Inpatient and outpatient diagnoses are recorded as International Classification of Diseases (ninth and tenth revisions) Clinical Modification (ICD-9/10-CM) codes. Data include inpatient and outpatient procedures, dates of service, retail and mail-order claims, inpatient stay, and provider specialty. Amounts allowed and paid by health plans are available for all services provided along with the dates of service for all claims. Other data elements available include demographic information (patient age, gender, and geographical region), product type (e.g., health maintenance organization and preferred provider organizations), payer type (e.g., commercial and self-insured), and start and stop dates of health plan enrollment.

Patients were selected for the study if they were treated with an AC^+^FV AVHC or a non-antistatic VHC (control VHC) between January 1, 2010 and August 31, 2015 (the “selection window”). The date of the first VHC device within the selection window was defined as the index date. All patients had to be treated with a long-term controller medication [inhaled corticosteroid (ICS) or fixed-dose combination of ICS and long-acting β2 agonist (ICS/LABA)] MDI within 60 days before or 60 days after the index date. All patients had at least 12 months of continuous healthcare coverage before the index date (“preindex period”), were diagnosed with asthma (ICD-9: 493.x) on index date or during the preindex period, and had at least 30 days of continuous healthcare coverage after the index date (“post-index period”). Patients were excluded if they had incomplete age, gender, or payer information; had a diagnosis of COPD (ICD-9: 490.x, 491.x, 492.x, 494.x, 495.x, and 496.x) on the index date or during the preindex period; or discontinued their long-term control MDI before index. In order to ensure that patients were newly treated with an AC^+^FV AVHC or control VHC, patients treated with AC^+^FV AVHC were excluded if they had evidence of non-AC^+^FV AVHC use (antistatic or control VHC) at any time during the study or AC^+^FV AVHC use during the preindex period. Patients treated with control VHC were excluded if they had evidence of any antistatic VHC use at any time during the study, or had control VHC use during the preindex period. Patients were categorized into mutually exclusive cohorts (AC^+^FV AVHC cohort or control VHC cohort) based on the type of VHC at the index date.

In order to reduce pretreatment confounders between the cohorts, patients in the AC^+^FV AVHC cohort were propensity score (PS) matched to patients in the control VHC cohort at a 1:1 ratio using a greedy matching algorithm without replacement [18]. Patients were matched on baseline age categories (0–2, 3–5, 6–12, 13–17, 18–34, 35–44, 45–54, 55–64, 65–74, 75 or older), gender, geographic region (Northeast, Midwest, South, West), Charlson comorbidity index (CCI) category (0, 1, 2, 3, 4+) [19], comorbid conditions [attention deficit hyperactivity disorder (ADHD), allergic rhinitis, anemia, anxiety, cancer, bronchopneumonia, cardiac disease, cerebrovascular disease, depression, diabetes, hypertension, obesity, other vascular diseases, pneumonia, pulmonary hypertension, and respiratory infection], medication history [ICS, short-acting β2 agonist (SABA), LABA, fixed-dose ICS/LABA and oral corticosteroids (OCS)], type of MDI (ICS or fixed-dose ICS/LABA), history of respiratory support including oxygen therapy and mechanical ventilation, history of severe exacerbations, history of moderate exacerbations, and history of all-cause hospitalizations. Patients without appropriate matches were not included in the analysis.

Treatment outcomes included number of patients with asthma exacerbations (moderate to severe); number of exacerbations per patient at 1, 6, 9, and 12 months after the index date; and time (days) to the first exacerbations. These measures were reported for patients with at least 12 months of post-index follow-up. Exacerbation severity was defined according to the literature [20–22]. A moderate exacerbation was defined as an ED visit claim (not leading to a hospital admission) with an asthma diagnosis, or an OCS prescription fill within 30 days of a physician visit with an asthma diagnosis; a severe exacerbation was defined as an inpatient admission claim with an asthma diagnosis. Furthermore, the incidence rate (IR) of exacerbations (either moderate or severe) was measured for patients with at least 30 days of post-index follow-up. The IR was defined as the number of patients with an event divided by total time spent at risk, which was from the index date to the end of enrollment, the end of the study period, discontinuation of index treatment (at least 60 days between the end of the supply and the next prescription fill), or the occurrence of an event—whichever occurred first during the variable follow-up. Asthma-related healthcare resource use measures included hospitalizations, outpatient visits, ED visits, laboratory tests, and ancillary and other services based on medical claims with an asthma diagnosis as well as prescriptions for an asthma medication. Asthma-related costs associated with these resources were also reported. Healthcare resource use and cost measures over 12 months of follow-up were reported for patients with at least 12 months of post-index follow up. The use of a fixed follow-up period was required to ensure a fair comparison of these measures between the two cohorts.

Baseline patient characteristics were reported using descriptive statistics, with frequency (*n*, %) for categorical measures and mean, standard deviation (SD), median, minimum, and maximum values for continuous variables. Treatment outcomes, healthcare resource use measures, and related costs in the AC^+^FV AVHC cohort and matched control VHC cohort were compared using bivariate chi-square tests for proportions and Student’s *t* test for means. The numbers of moderate-to-severe exacerbations per patient in the AC^+^FV AVHC and control VHC cohorts were also compared using rate ratios, defined as the number of moderate-to-severe exacerbations per patient in the antistatic VHC cohort divided by the number of moderate-to-severe exacerbations per patient in the control VHC cohort. The proportions of patients without an exacerbation in the AC^+^FV AVHC cohort and the control VHC cohort were compared using a Kaplan–Meier estimation of time to first occurrence of a moderate-to-severe exacerbation. In addition to the* t* test of differences in the mean costs of the two matched sample, a regression analysis was performed in the unmatched population to confirm the results of the bivariate analysis of healthcare costs in the matched population. In the regression analysis, the marginal effect of AC^+^FV AVHC on per-patient costs for moderate-to-severe exacerbations was measured using generalized linear models (GLMs) with a log link and gamma family distribution, along with covariate adjustments to control for potential confounders. All inferential statistical analyses were conducted assuming a two-tailed test of significance and a alpha level set a priori at 0.05. All analyses were conducted using SAS 9.4 (Cary, NC, USA).

No institutional review board (IRB) review was required for this retrospective cohort analysis using HIPAA-compliant de-identified patient data.

## Results

### Demographics and Clinical Characteristics

There were 156,801 patients treated with the AC^+^FV AVHC in the database within the selection window. After applying the selection criteria, 13,995 antistatic VHC patients were retained. After PS matching, a total of 9325 antistatic VHC patients and 9325 control VHC patients were identified for the analyses (Table 1).

**Table 1:** Selection of patients for the AC^+^FV AVHC cohort

Selection criteria	Excluded		Remaining	
	*N*	%	*N*	%
Inclusion criteria				
Evidence of the AC^+^FV AVHC between January 1, 2010 through August 31, 2015 (the date of first antistatic VHC was defined as the index date)	–	–	156,801	100.0
Evidence of MDI use 60 days before or after index AC^+^FV AVHC claim	111,672	71.2	45,129	28.8
Continuous enrollment for ≥30 days after index date (post-index follow-up was variable)	3515	2.2	41,614	26.5
Meeting requirements for continuous enrollment before index date (preindex period)	11,532	7.4	30,082	19.2
≥1 diagnosis of asthma during the preindex period or on index date	9111	5.8	20,971	13.4
Exclusion criteria				
Incomplete data (age, gender, payer)	13	0.1	20,958	13.4
Evidence of any VHC other than the AC^+^FV AVHC use at any time during the study period	4548	2.9	16,410	10.5
Evidence of any antistatic VHC use in the preindex period	177	0.1	16,233	10.4
Diagnosis of COPD during the preindex period or on the index date	1930	1.2	14,303	9.1
Discontinuation of long-term control MDI before index	348	0.2	13,955	8.9

Table 2 shows baseline demographic and clinical characteristics for the AC^+^FV AVHC and control VHC cohorts. The cohorts were balanced on all baseline characteristics (*p* values >0.05). The mean age was 12.2 years, and the majority of patients were 17 years or younger. All patients had at least one comorbidity (CCI score ≥1), and the most common comorbidities were respiratory infection (≥68.7%), allergic rhinitis (≥40.3%), and pneumonia (≥12.4%). The majority of the patients were prescribed an ICS (≥87.1%) and/or SABA (≥85.3%) on or prior to the index VHC, and half of the patients were prescribed an OCS (≥50.3%). Most patients used their index VHC with an ICS (≥93.0%). Prior to the index VHC, less than half of the patients had a moderate exacerbation (≥45.0%), and fewer had a severe exacerbation (≥4.4%) or an all-cause hospitalization (≥6.0%). Very few patients were on oxygen therapy or mechanical ventilation (≥0.2%).

**Table 2:** Baseline characteristics

Demographic and clinical characteristics	AC^+^FV AVHC		Control VHC		*p* value
	*N* = 9325		*N* = 9325		
	*N*	(%)	*N*	(%)	
Age on index date (years)					
Mean ± SD	12.23	± 13.65	12.24	± 13.75	0.7405
Median	8		8		
Age category (years)					
0–2	579	6.2%	602	6.5%	0.1577
3–5	2326	24.9%	2324	24.9%	0.9347
6–12	4115	44.1%	4124	44.2%	0.7517
13–17	983	10.5%	970	10.4%	0.5903
18–34	465	5.0%	461	4.9%	0.8453
35–44	270	2.9%	246	2.6%	0.1783
45–64	282	3.0%	283	3.0%	0.9573
55–64	230	2.5%	239	2.6%	0.6280
65–74	64	0.7%	57	0.6%	0.5175
75 or older	11	0.1%	19	0.2%	0.1167
0–17	8003	85.8%	8020	86.0%	0.6021
18 or older	1322	14.2%	1305	14.0%	0.6021
Gender					
Female	4134	44.3%	4101	44.0%	0.3369
Male	5191	55.7%	5224	56.0%	0.3369
Geographic region					
Northeast	3477	37.30%	3460	37.10%	0.1693
West	2430	26.10%	2468	26.50%	0.1462
Midwest	1907	20.50%	1914	20.50%	0.7876
South	1511	16.20%	1483	15.90%	0.0695
Charlson comorbidity index (CCI)^#^					
Mean ± SD	1.05	± 0.36	1.05	± 0.33	0.5829
Median	1		1		
0	0	0.0%	0	0.0%	NA
1	9021	96.7%	9031	96.8%	0.6604
2	176	1.9%	172	1.8%	0.8214
3	86	0.9%	85	0.9%	0.9372
4+	42	0.5%	37	0.4%	0.5737
Comorbid conditions					
Respiratory infection	6411	68.80%	6405	68.70%	0.8585
Allergic rhinitis	3762	40.30%	3771	40.40%	0.7901
Pneumonia	1154	12.40%	1167	12.50%	0.6427
Anxiety	436	4.70%	404	4.30%	0.1613
ADHD	428	4.60%	416	4.50%	0.5699
Obesity	378	4.10%	314	3.40%	0.0041
Depression	304	3.30%	277	3.00%	0.2076
Hypertension	294	3.20%	286	3.10%	0.7061
Cardiac disease	246	2.60%	207	2.20%	0.0538
Anemia	215	2.30%	192	2.10%	0.1878
Other vascular diseases	177	1.90%	154	1.70%	0.1820
Cancer	165	1.80%	145	1.60%	0.2418
Diabetes	110	1.20%	115	1.20%	0.7295
Cerebrovascular disease	38	0.40%	40	0.40%	0.8185
Pulmonary hypertension	12	0.10%	7	0.10%	0.2513
Asthma medication history					
ICS	8118	87.10%	8118	87.10%	1.000
SABA	7953	85.30%	7953	85.30%	1.000
OCS	4754	51.00%	4693	50.30%	0.0784
Fixed-dose (LABA/ICS) combination	684	7.30%	684	7.30%	1.000
LABA	17	0.20%	19	0.20%	0.7389
Index MDI (including 60 days post-index)					
ICS	8690	93.2%	8676	93.0%	0.3293
Fixed-dose (LABA/ICS) combination	635	6.8%	649	7.0%	0.3293
History of respiratory support					
Oxygen therapy/mechanical ventilation	23	0.2%	29	0.3%	0.3961
History of severe exacerbation	433	4.6%	408	4.4%	0.2443
History of moderate exacerbation	4255	45.6%	4197	45.0%	0.0891
History of any-cause hospitalization	597	6.4%	562	6.0%	0.1668

### Occurrence of Moderate-to-Severe Exacerbations

**Fig. 1:**
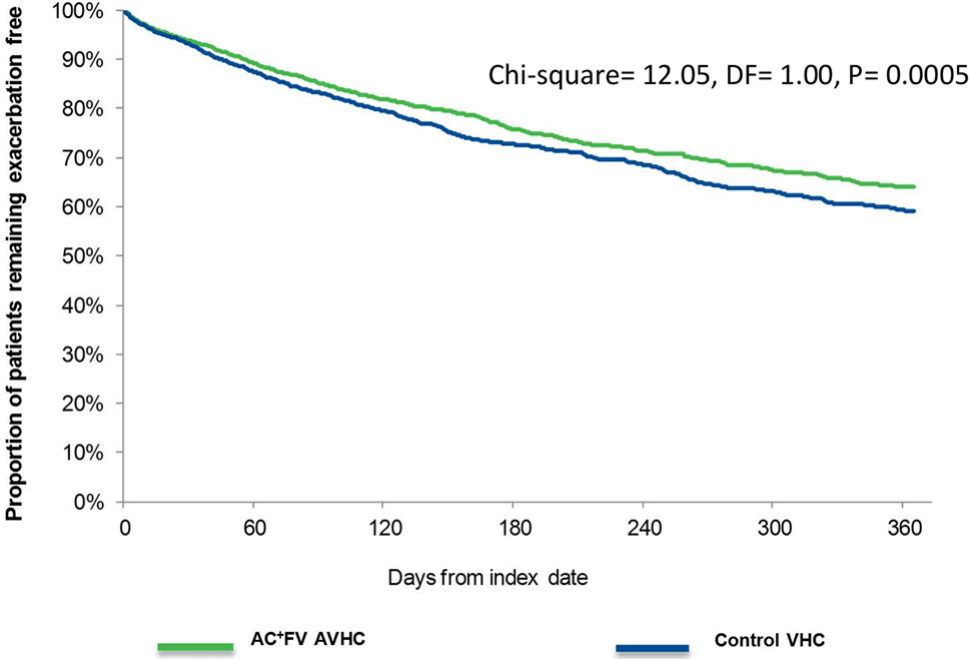
Kaplan–Meier curve for time to exacerbation

Among patients with at least 30 days of follow-up (*n* = 9325 for each cohort), the incidence rate of moderate-to-severe exacerbations per 100 person-days (95% CI) was significantly higher in the control VHC cohort than in the AC^+^FV AVHC cohort [0.161 (0.150–0.172) vs. 0.137 (0.128–0.147)] (Table 3). Average time from the index date to the first moderate-to-severe exacerbation was 141.9 (SD ±106.87) days for the AC^+^FV AVHC cohort and 135.7 (SD ±107.62) days for the control VHC cohort (*p* = 0.2714). The Kaplan–Meier curve of time to moderate-to-severe exacerbation revealed that the proportion of exacerbation-free patients was greater in the AC^+^FV AVHC cohort than the control VHC cohort throughout the study period (chi square = 12.05, *DF* = 1.00, *p* < 0.05; Fig. 1).

**Table 3:** Incidence rates of moderate or severe exacerbations for patients with at least 30 days of follow-up

Treatment group	Number of patients with a moderate or severe exacerbation	Total person days at risk	Incidence rate	95% CI
AC^+^FV AVHC (*N* = 9325)	1160	737,392	0.14	[0.128, 0.147]
Control VHC (*N* = 9325)	1292	710,269	0.16	[0.150, 0.172]

**Fig. 2:**
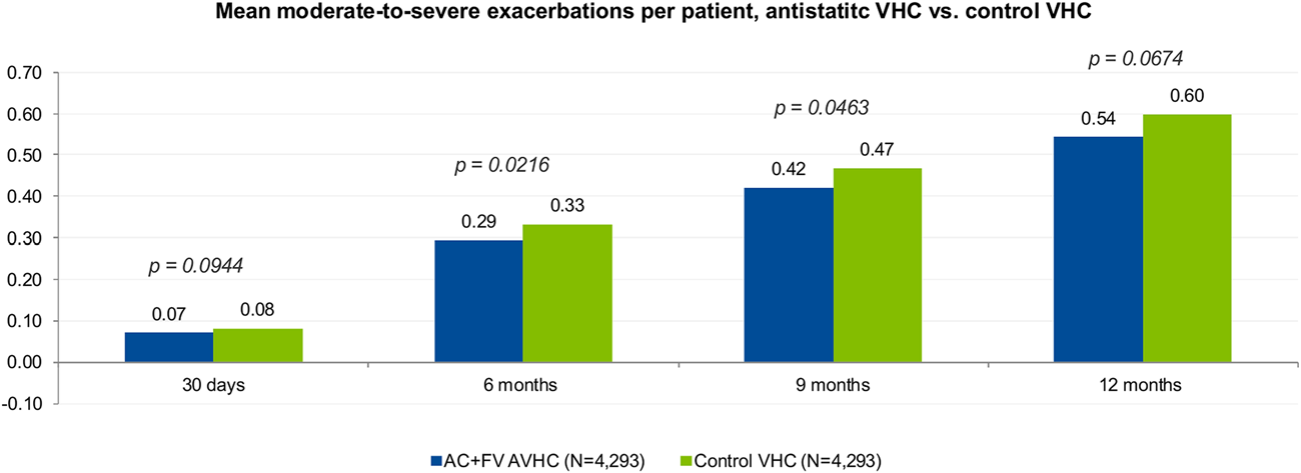
Moderate-to-severe exacerbations per patient at 30 days, 6 months, 9 months, and 12 months

Table 4 shows the occurrence of moderate-to-severe exacerbations among the subgroup of patients with at least 12 months of follow-up. Among the 4293 patients in each cohort, 29.5% of the AC^+^FV AVHC patients (*n* = 1265) and 30.6% of the control VHC patients (*n* = 1314) had at least one moderate-to-severe exacerbation during the study period (*p* = 0.2487). Mean number of moderate-to-severe exacerbations per patient at 1 month was 0.07 (SD ±0.34) for AC^+^FV AVHC and 0.08 (SD ±0.34) for control VHC (*p* = 0.0944); mean number of moderate-to-severe exacerbations per patient at 12 months was 0.54 (SD ±1.17) for AC^+^FV AVHC and 0.60 (SD ±1.30) for control VHC (*p* = 0.0674). Patients using the AC^+^FV AVHC had a significantly lower number of moderate-to-severe exacerbations compared to those using the control VHC both at 6 months [0.29 (SD ±0.78) for AC^+^FV AVHC and 0.33 (SD ±0.86) for control VHC; *p* < 0.05] and at 9 months [0.42 (SD ±0.99] for AC^+^FV AVHC and 0.47 (SD ±1.09) for control VHC; *p* < 0.05]. The rate ratios throughout the 12-month follow-up period (0.88–0.90) consistently showed that patients with AC^+^FV AVHC use were 10–12% less likely to experience a moderate-to-severe exacerbation compared to patients with control VHC use. The mean number of moderate-to-severe exacerbations per patient at each time point in each cohort is shown in Fig. 2.

**Table 4:** Occurrence of exacerbations (moderate to severe) among patients with at least 12 months of follow-up

Occurrence of exacerbation	AC^+^FV AVHC	Control VHC	Rate ratio	*p* value
	*N* = 4293	*N* = 4293		
Patients with at least one exacerbation				
*n* (%)	1265 (29.5)%	1314 (30.6)%	–	0.2487
Exacerbations per patient (mean ± SD)				
At 30 days	0.07 ± 0.34	0.08 ± 0.34	0.88	0.0944
At 6 months	0.29 ± 0.78	0.33 ± 0.86	0.88	0.0216
At 9 months	0.42 ± 0.99	0.47 ± 1.09	0.90	0.0463
At 12 months	0.54 ± 1.17	0.60 ± 1.30	0.90	0.0674
Time to exacerbation (days)				
Mean ± SD	141.9 ± 106.87	135.7 ± 107.62	–	0.2714

### Asthma-Related Healthcare Resource Use

In Table 5 it can be seen that, over 12 months of follow-up, a small proportion of the patients had an asthma-related hospitalization in both cohorts (3.0% for AC^+^FV AVHC vs. 3.7% for control VHC; *p* = 0.0702). The average number of hospitalizations per patient was 0.04 for both cohorts (*p* = 0.2041). The majority of the patients visited a doctor’s office (≥86.8%; *p* = 0.7990) at a rate of ≥2.7 visits per patient (*p* = 0.2775). The proportion of patients visiting the ED was significantly lower in the AC^+^FV AVHC cohort than in the control VHC cohort (10.8% vs. 12.4%; *p* < 0.05), and the number of ED visits per patient was also significantly lower in the AC^+^FV AVHC cohort compared to the control VHC cohort (0.15 visits vs. 0.18 visits; *p* < 0.05). All patients in both groups had at least one asthma-related pharmacy fill, with 6.9 fills per patient for the AC^+^FV AVHC cohort and 7.1 fills per patient for the control VHC cohort (*p* = 0.2350).

**Table 5:** Healthcare resource use

Utilization measures	AC^+^FV AVHC	Control VHC	*p* value
	*N* = 4293	*N* = 4293	
Hospitalizations			
Patients with at least 1 hospitalization (*n*, %)	127 (3.0)%	157 (3.7)%	0.0702
Hospitalizations per patient (mean, SD)	0.04 ± 0.22	0.04 ± 0.23	0.2041
Outpatient visits			
Patients with at least 1 outpatient visit (*n*, %)	3720 (86.7)%	3728 (86.8)%	0.7990
Outpatient visits per patient (mean, SD)	2.67 ± 3.17	2.75 ± 3.27	0.2775
ED visits			
Patients with at least 1 ED visit (*n*, %)	462 (10.8)%	533 (12.4)%	0.0167
ED visits per patient (mean, SD)	0.15 ± 0.51	0.18 ± 0.58	0.0026
Pharmacy fills			
Patients with at least 1 pharmacy fill (*n*, %)	4293 (100.0)%	4293 (100.0)%	NA
Fills per patient (mean, SD)	6.91 ± 4.62	7.08 ± 4.80	0.2350

### Moderate-to-Severe Exacerbation-Related Cost

The per patient cost associated with a moderate-to-severe exacerbation for the AC^+^FV AVHC patients and the control VHC patients was $78.39 (SD ±$1769.38) and $68.17 (SD $±1057.83; *p* = 0.1976) at 30 days, $259.96 (SD ±$3116.38) and $321.16 (SD ±$4704.31; *p* = 0.0983) at 6 months, $393.01 (SD ±$3969.09) and $444.60 (SD ±$5003.96; *p* = 0.1391) at 9 months, and $514.25 (SD ±$4259.03) and $544.07 (SD ±$5257.33; *p* = 0.1455) at 12 months (Table 6).

**Table 6:** Moderate-to-severe exacerbation costs

Cost per patient	AC^+^FV AVHC	Control VHC	*p* value
	*N* = 4293	*N* = 4293	
Moderate-to-severe exacerbation cost per patient (mean ± SD)			
At 30 days	$78.39 ± $1769.38	$68.17 ± $1057.83	0.1976
At 6 months	$259.96 ± $3116.38	$321.16 ± $4704.31	0.0983
At 9 months	$393.01 ± $3969.09	$444.60 ± $5003.96	0.1391
At 12 months	$514.25 ± $4259.03	$544.07 ± $5257.33	0.1455

In the regression model, the cost of treating a moderate-to-severe exacerbation was found to be significantly lower for the AC^+^FV AVHC patients than for the control VHC patients at 30 days (23% lower; *p* < 0.05), at 6 months (25% lower; *p* < 0.05), at 9 months (20% lower; *p* < 0.05), and at 12 months (12% lower; *p* < 0.05) (Table 7).

**Table 7:** Marginal effect of AC^+^FV AVHC on exacerbation-related costs

Parameter	Parameter estimate	Exp (b)	Standard error	*p* value
Cost of exacerbation in the AC^+^FV AVHC group at 30 days from index date	−0.26	0.77	0.04	<0.0001
Reference: control VHC group				
Cost of exacerbation in the AC^+^FV AVHC group at 6 months from index date	−0.29	0.75	0.04	<0.0001
Reference: control VHC group				
Cost of exacerbation in the AC^+^FV AVHC group at 9 months from index date	−0.23	0.80	0.04	<0.0001
Reference: control VHC group				
Cost of exacerbation in the AC^+^FV AVHC group at 12 months from index date	−0.13	0.88	0.04	0.0017
Reference: control VHC group				

## Discussion

This is the first real-world study comparing an AC^+^FV AVHC with control non-antistatic VHCs. Findings from this study add to the body of literature supporting the benefits of using VHCs with inhaler medications for treating asthma [23, 24]. The addition of VHCs can help make hand-held inhalers easier to use, especially for patients who have difficulty using MDIs. Asthma disease control is highly associated with drug delivery to the lungs, and the use of VHCs can help improve deposition of asthma medications into the lower respiratory tract compared to MDIs alone. Moreover, an antistatic VHC could result in better drug delivery compared to non-antistatic VHCs by limiting the electrostatic charge in the chamber and increasing the amount of aerosol cloud available for deposition to the lungs [25].

In our study, about 29.5% of the patients in the AC^+^FV AVHC group experienced at least one moderate-to-severe exacerbation in the 12-month follow-up period (30.6% of patients in the control VHC group), which is slightly lower than the proportion of patients with an exacerbation (34.4%) reported in a previously published retrospective database study conducted in a similar patient population [26]. That study also reported a mean of 1.40 exacerbations per patient per year in females and a mean of 1.43 exacerbations per patient per year in males on ICS therapy, as well as 1.42 exacerbations per patient per year in females and 1.40 exacerbations per patient per year in males on ICS/LABA therapy [26]. In comparison, our study reports fewer exacerbations per patient (0.54 moderate-to-severe exacerbations per patient in the AC^+^FV AVHC group at 12 months and 0.60 moderate-to-severe exacerbations per patient in the control VHC group at 12 months). The rate ratios of exacerbations between the two cohorts show that the likelihood of patients in the AC^+^FV AVHC group experiencing a moderate-to-severe exacerbation trends lower than that for the control VHC group throughout the follow-up period, suggesting that adding the AC^+^FV AVHC to an MDI may help improve the management of asthma exacerbations compared to adding a control VHC.

Additionally, among patients with at least 30 days of follow-up, we found that the AC^+^FV AVHC group had a significantly lower incidence rate of moderate-to-severe exacerbations than the control VHC group. This has potential implications for early treatment outcomes in asthma such as controlling future exacerbations because previous exacerbations have been reported to be a risk factor for future exacerbations [27, 28]. The difference in exacerbation trends, which was sustained over 12 months according to the Kaplan–Meier analysis, suggests that patients using the AC^+^FV AVHC may experience better and sustained outcomes than those using a control VHC type. Since exacerbations are acute events which often require unscheduled healthcare resource utilization, the utilization of ED following treatment initiation can be used as a surrogate marker for assessing the effect of treatment on the occurrence of exacerbations [29]. Our analysis demonstrated that the rate of ED visits per patient in the AC^+^FV AVHC group was significantly lower than that in the control VHC group in the follow-up. This finding supports the additional study observation that exacerbation events in the AC^+^FV AVHC group were consistently lower than in the control VHC group in real-world data. The lower occurrence of asthma-related ED visits observed in the AC^+^FV AVHC group may lead to an expectation that the proportion of patients with an asthma-related hospitalization would also be smaller in the AC^+^FV AVHC group compared to the control group. Nonetheless, despite the slightly lower proportion observed (3.0% vs. 3.7%), the differences did not reach a statistically significant level.

Exacerbations are associated with high healthcare resource use and consequently the high economic burden of asthma [29]. An administrative claims database study found that patients with exacerbations had a mean per patient healthcare cost of $4212 more than those without an exacerbation [20]. Therefore, controlling exacerbations is important. No significant differences in the cost associated with a moderate-to-severe exacerbation were observed between AC^+^FV AVHC patients and control VHC patients when the mean costs were compared. This could be partly the result of the high degree of skewness in the cost data. Our alternative analysis using the regression model in the unmatched cohorts, adjusting for potential confounders and addressing the issues of outliers and heavy-tailed cost data, however, showed statistically significantly lower exacerbation-related costs associated with the AC^+^FV AVHC compared to the control VHC at 30 days, 6 months, 9 months, and 12 months. This finding suggests that the AC^+^FV AVHC may well help reduce exacerbation-related costs when compared to control VHCs.

As mentioned in the “Introduction,” there are laboratory studies which support the hypothesis that, in a real-world setting, medication delivery via an antistatic VHC would be closer to optimal and more consistent than delivery via a non-antistatic VHC. Our study appears to provide some real-world validation of that hypothesis in terms of asthma control; however, we recognize that the presence of the inhalation feedback indicator in the AC^+^FV AVHC may also have contributed to the observed outcome differences.

There are inherent limitations to the use of secondary data and observational study methods that should be considered when interpreting results. The adjudicated claims database does not report clinical disease information (e.g., lung function, symptoms, etc.) and it was not possible to define severity by clinical markers; patients were diagnosed using ICD codes created for billing. Training for the proper use of the device from providers as well as adherence to device use is important for attaining benefits from devices; however, this information was not available in our database. The database contains commercially insured patients and our findings may not be generalizable to the Medicare or Medicaid population.

There are, however, several strengths of our study. Since the QuintilesIMS Real-World Data Adjudicated Claims Database is representative of the US commercially insured population, the results from our study are generalizable to the commercially insured US population. The cost and utilization analyses performed in our study involved fully adjudicated claims data on healthcare services (hospitalizations, ED, outpatient, pharmacy, and other services) and all associated costs. The study also employed PS matching, which addressed the issue of multicollinearity in observational studies, since covariates may be highly correlated (e.g., age and comorbidities).

## Conclusions

The AeroChamber Plus^®^ Flow Vu^®^ Antistatic VHC was associated with delayed time to first exacerbation, a lower occurrence of asthma-related ED visits, and lower exacerbation-related costs when compared to control non-antistatic VHCs. Exacerbation rates also trended lower for the AC^+^FV AVHC throughout the 12-month study period. Findings from our study therefore suggest that the use of the AC^+^FV AVHC in association with a pMDI may result in better asthma control compared to the use of non-antistatic VHCs.
